# Developing and Evaluating Large Language Model–Generated Emergency Medicine Handoff Notes

**DOI:** 10.1001/jamanetworkopen.2024.48723

**Published:** 2024-12-03

**Authors:** Vince Hartman, Xinyuan Zhang, Ritika Poddar, Matthew McCarty, Alexander Fortenko, Evan Sholle, Rahul Sharma, Thomas Campion, Peter A. D. Steel

**Affiliations:** 1 Abstractive Health, New York, New York; 2 Department of Emergency Medicine, NewYork-Presbyterian/Weill Cornell Medicine, New York; 3 Department of Population Health, NewYork-Presbyterian/Weill Cornell Medicine, New York; 4 Clinical and Translational Science Center, Weill Cornell Medicine, New York, New York

## Abstract

**Importance:**

An emergency medicine (EM) handoff note generated by a large language model (LLM) has the potential to reduce physician documentation burden without compromising the safety of EM-to-inpatient (IP) handoffs.

**Objective:**

To develop LLM-generated EM-to-IP handoff notes and evaluate their accuracy and safety compared with physician-written notes.

**Design, Setting, and Participants:**

This cohort study used EM patient medical records with acute hospital admissions that occurred in 2023 at NewYork-Presbyterian/Weill Cornell Medical Center. A customized clinical LLM pipeline was trained, tested, and evaluated to generate templated EM-to-IP handoff notes. Using both conventional automated methods (ie, recall-oriented understudy for gisting evaluation [ROUGE], bidirectional encoder representations from transformers score [BERTScore], and source chunking approach for large-scale inconsistency evaluation [SCALE]) and a novel patient safety-focused framework, LLM-generated handoff notes vs physician-written notes were compared. Data were analyzed from October 2023 to March 2024.

**Exposure:**

LLM-generated EM handoff notes.

**Main Outcomes and Measures:**

LLM-generated handoff notes were evaluated for (1) lexical similarity with respect to physician-written notes using ROUGE and BERTScore; (2) fidelity with respect to source notes using SCALE; and (3) readability, completeness, curation, correctness, usefulness, and implications for patient safety using a novel framework.

**Results:**

In this study of 1600 EM patient records (832 [52%] female and mean [SD] age of 59.9 [18.9] years), LLM-generated handoff notes, compared with physician-written ones, had higher ROUGE (0.322 vs 0.088), BERTScore (0.859 vs 0.796), and SCALE scores (0.691 vs 0.456), indicating the LLM-generated summaries exhibited greater similarity and more detail. As reviewed by 3 board-certified EM physicians, a subsample of 50 LLM-generated summaries had a mean (SD) usefulness score of 4.04 (0.86) out of 5 (compared with 4.36 [0.71] for physician-written) and mean (SD) patient safety scores of 4.06 (0.86) out of 5 (compared with 4.50 [0.56] for physician-written). None of the LLM-generated summaries were classified as a critical patient safety risk.

**Conclusions and Relevance:**

In this cohort study of 1600 EM patient medical records, LLM-generated EM-to-IP handoff notes were determined superior compared with physician-written summaries via conventional automated evaluation methods, but marginally inferior in usefulness and safety via a novel evaluation framework. This study suggests the importance of a physician-in-loop implementation design for this model and demonstrates an effective strategy to measure preimplementation patient safety of LLM models.

## Key Points

**Question:**

Can a large language model (LLM) generate emergency medicine (EM)-to-inpatient (IP) handoff notes that are useful and safe for EM care?

**Findings:**

In this cohort study of 1600 EM patient medical records using a novel evaluation framework, the LLM-generated EM-to-IP handoff notes had a mean usefulness of 4.04 out of 5 (compared with 4.36 for physician-written) and a mean patient safety of 4.06 out of 5 (compared with 4.50 for physician-written) with no critical patient safety risks.

**Meaning:**

These findings suggest the value of a manual, patient safety–focused clinical evaluation of LLM models and the potential of LLM-generated handoff notes to create a new standard of care in EM.

## Teaser

This cohort study evaluates large language model (LLM)–generated emergency medicine–to-inpatient handoff notes and evaluates their accuracy and safety compared with physician-written notes.

## Introduction

Handoffs, where patient information is exchanged between health professionals during a transfer of clinical responsibility, have been identified as a critical source of medical errors.^1,2^ The Joint Commission, the Accreditation Council for Graduate Medical Education, and the Association of American Medical Colleges have all recommended the development of high-quality and standardized handoff processes to address the substantial patient risk of this ubiquitous event.^3,4^ Implementing handoff tools has previously demonstrated significant reductions in medical errors.^5,6^ High-quality handoffs from emergency medicine (EM) to inpatient (IP) services (EM-to-IP) are challenged by medical complexity, diagnostic uncertainty, rapidly evolving care plans, and time constraints.^7,8,9,10^ The EM-to-IP handoff structure is not well standardized, frequently communicated verbally, and poorly adhered to in emergency departments (EDs), including in medical centers with formalized handoff systems.^11,12,13,14^ Prior research has demonstrated that suboptimal EM-to-IP handoff is associated with adverse events, EM leaders and front-line clinicians themselves view the EM-to-IP handoff as high risk, and an electronic health record (EHR)-based technology is commonly mentioned as the most desired assistive tool in improving ED transitions of care.^15,16,17,18^ Limited work to date has demonstrated EM electronic handoff tools as feasible, efficient, and effective.^19,20,21^ In April 2023, EM and internal medicine leadership of the study site collaboratively developed and launched a mandatory, EHR-based handoff workflow via a standardized EM-to-IP handoff note template, designed for real-time completion by the EM care team at time of admission. At 3 and 6 months postlaunch, informal evaluation of new EM-to-IP handoff notes through random medical record review and unstructured clinician feedback sessions revealed variable completeness, quality, and subsequent usefulness of the handoff notes.

In recent years there has been an accelerated interest in using LLMs to automate clinical tasks in an effort to unburden physicians and reduce burnout.^22^ Computer-generated text within clinical notes using natural language processing (NLP) have been overall shown to improve note completion rates, physician satisfaction, and patient outcomes.^23^ Since 2018, NLP has made rapid advancements in health care with the discovery of the transformer model architecture, the building block of large language models (LLMs). LLMs can automate workflows such as discharge summaries,^24^ radiology reports,^25^ patient messaging,^26^ after-visit summaries,^27^ and ambient dictation^28^ with various levels of perceived quality in each workflow.^29^ LLMs are particularly effective at summarizing large unstructured clinical datasets, such as ED patient medical records.^30^ A common concern of LLMs is their ability to hallucinate data, or LLMs generating output text that is not factually consistent with the original source content.^31^ Much work has been done in health care to reduce hallucinations through building larger-parameter models trained on trillions of datasets, and then instruction fine-tuning the LLM on smaller, well-curated datasets.^32,33^ LLMs can also be designed with explainability by citing inferred content back to the reference source notes.^34^ For short-context length notes, using few-shot prompt engineering approaches with large language models like GPT-4 can produce summaries that outperform standard physician documentation in completeness and error frequency.^35^ However, factual inconsistencies in the summaries produced by LLMs increase as the context length increases,^36^ and for medium- to long-context tasks, fine-tuning an open-source model has been shown to perform better than a prompt-learning approach.^37^ In prior work, members of this study team demonstrated 62% of LLM-generated hospital course summaries met standard-of-care for a formal inpatient discharge summary.^24^ However, recently published clinical evaluation frameworks may not address the anticipated effect LLM performance limitations could have on patient safety.^38,39,40,41^

In this study, we aim to expand on prior work of clinical summarization to rigorously evaluate the outcomes of a fine-tuned model developed to generate accurate and safe summaries of the care rendered during an ED visit, with the long-term goal of integrating automated, structured EM-to-IP handoff notes into an EHR-based electronic handoff admission workflow (see eAppendix 1 in Supplement 1). We fine-tune pretrained LLMs on well curated datasets of structured and unstructured EHR data from the ED encounter to summarize the patient’s ED care. We improved the correctness of model generations and customized the summaries in a structured format designed by a team of EM and internal medicine physician leaders for optimal usefulness. We proposed a novel patient safety-focused LLM evaluation framework to examine the LLM-generated handoff notes’ quality and accuracy and the downstream patient safety implications of any identified inaccuracies. To evaluate noninferiority, we compared the LLM-generated handoff notes with the preexisting physician-written EM-to-IP handoff notes as the active control, using both the proposed patient safety-focused clinical evaluation framework and automated benchmark-driven methods. We used the physician-written EM-to-IP handoff notes as the active control and used the scores from both evaluation frameworks for the margin of inferiority of the intervention.

## Methods

### Data Collection

The study, with review and approval from the Weill Cornell institutional review board (IRB), was conducted at an urban academic 840-bed quaternary-care hospital in New York City, with approximately 71 000 adult ED visits and 21 000 admissions annually. EHR data from 1600 individual EM patient encounters leading to acute hospital admission were randomly selected from visits occurring between April and September of 2023. We limited our analysis to EM patient encounters occurring after April 2023, as the study site had updated the EM-handoff at that time. Encounters before this date used an earlier version of the EM-handoff note that would have provided suboptimal data for training labels. We used these data to fine-tune a pretrained LLM, which then generated an abstractive EM-handoff note. For the 1600 patient encounters (the study participants), Weill Cornell Medicine IRB approved a waiver of informed consent because the study used retrospective data and posed minimal risk to patients. We used Strengthening the Reporting of Observational Studies in Epidemiology (STROBE) reporting guidelines.

### EM-to-IP Handoff Note Template

The EM-to-IP handoff note template used in the study is a replication of the current manual handoff note structure used at the study site. The generated EM handoff note consists of components generated by a rule-based pattern-matching approach (laboratory tests, vitals, medications, consult orders, and radiology impressions) and components generated by the trained abstractive summarization model (history of present illness [HPI], differential diagnoses, immediate care plans, in-ED events, and disposition). Each summary also included a header with the timestamp of ED triage and discharge, patient’s birth date, patient’s unique identifier, patient’s encounter number, and the total time of patient’s stay in the ED.

### Data Curation for Automated ED Note Generation

The EHR data were bifurcated into 2 datasets linked by the patient encounter number: 1 for the rule-based pattern-matching approach and the other for the LLM fine-tuning discussed in further detail in eAppendix 1 in Supplement 1. The rule-based framework was designed by the 3 board certified EM physicians (M.M., A.F., and P.S.). Fine tuning of the pretrained LLM consisted of the notes in Table 1: EM clinician notes, consultation notes, EM progress note entries, and EM procedure notes. The EM-to-IP handoff notes were used as the labels. As the preexisting labels were of variable quality for LLM-model training, an informatics professional (V.H.) worked over a period of 200 hours with 3 board certified emergency medicine physician leaders with experience in formal quality and patient safety review processes (M.M., A.F., and P.S.) to improve the dataset through manual curation and annotation. As the task of EM-handoff note generation is not dependent on racial characteristics of the patients, we removed all mentions of race during the annotation stage as a means to avoid race bias; therefore, the model was trained to generate text without race-based assumptions. Although resource intensive, a small and carefully curated dataset of at least 1000 examples has been shown to be sufficient to produce remarkable results for the language model chosen.^42^ Given the size of our dataset, we created a train and test dataset with a ratio of 1500:100, with a higher ratio of data placed in the training set and eschewed a validation set to lower the variance of the models. We used k-fold cross validation on the training dataset to avoid sampling bias for the hyperparameter optimization of the LLMs.

**Table 1.** Types of Data Included From the Emergency Department (ED) Patient Electronic Health Record^a^

Type of data	Description
Descriptive	Date of birth, medical record number, encounter number, and total time of stay in ED
Encounter	ED arrival date and time, IP admit date and time
Laboratory tests (all results available)	Examples: hemoglobin, hematocrit, white blood cell count, neutrophil count, platelets, sodium, potassium, chloride, bicarbonate, creatinine, blood urea nitrogen, troponin, D dimer, lactate, urinalysis, ketone, blood, nitrite, leucocytes, and red blood cells
Laboratory tests (only if abnormal)	Examples: β-human chorionic gonadotropin hormone, all serum drug levels (alcohol level, salicylate level, Tylenol level), magnesium, lipase, and erythrocyte sedimentation rate
Notes (in order of hierarchy)	EM clinician notes, consultation notes, EM progress notes, and EM procedure notes
Vitals	Height, weight, temperature, heart rate, blood pressure, and peripheral capillary oxygen saturation
Orders	Medications, consults, and radiology results

Abbreviations: EM, emergency medicine; IP, inpatient.

^a^ Automated EM handoff notes are generated from the curation of the data through both rule-based and large language model–summarization approaches.

### Models

For this study, we chose the LLMs Robustly Optimized BERT Approach (RoBERTa; hereafter referred to as LLM 1)^43^ for saliency content selection and Large Language Model Meta AI 2 (Llama-2; hereafter referred to as LLM 2) 7B^44^ for abstractive summarization. Further information about the models and technology specifications is provided in detail in eAppendix 1 in Supplement 1.

### Data Processing

As LLM 2 only has a context size of 4096 tokens,^44^ we used 2 steps to process the EM notes to both shorten the input size while maintaining content salience. First, we adopted a number of heuristic strategies for prioritization and filtration: (1) clinical note types (hierarchy presented in Table 1), (2) time of authorship, and (3) duplicate sentence detection. Second, we used an LLM 1–based saliency model to infer EM note sentences based on likelihood of content contribution to the EM-to-IP handoff notes.

### Model Training and Inference

Our summarization model is a fine-tuned decoder-only causal language model based on LLM 2. We used different prompts for the separate types of summarization: HPI and EM handoff. Additional information about the model training and inference process is provided in eAppendix 1 in Supplement 1.

Using a combination of generative AI powered by our fine-tuned LLM 2 model and a set of heuristic rules, our summarization system produced ED handoff notes with various sections for downstream clinical tasks. The inference process is shown in the Figure.

**Figure.**
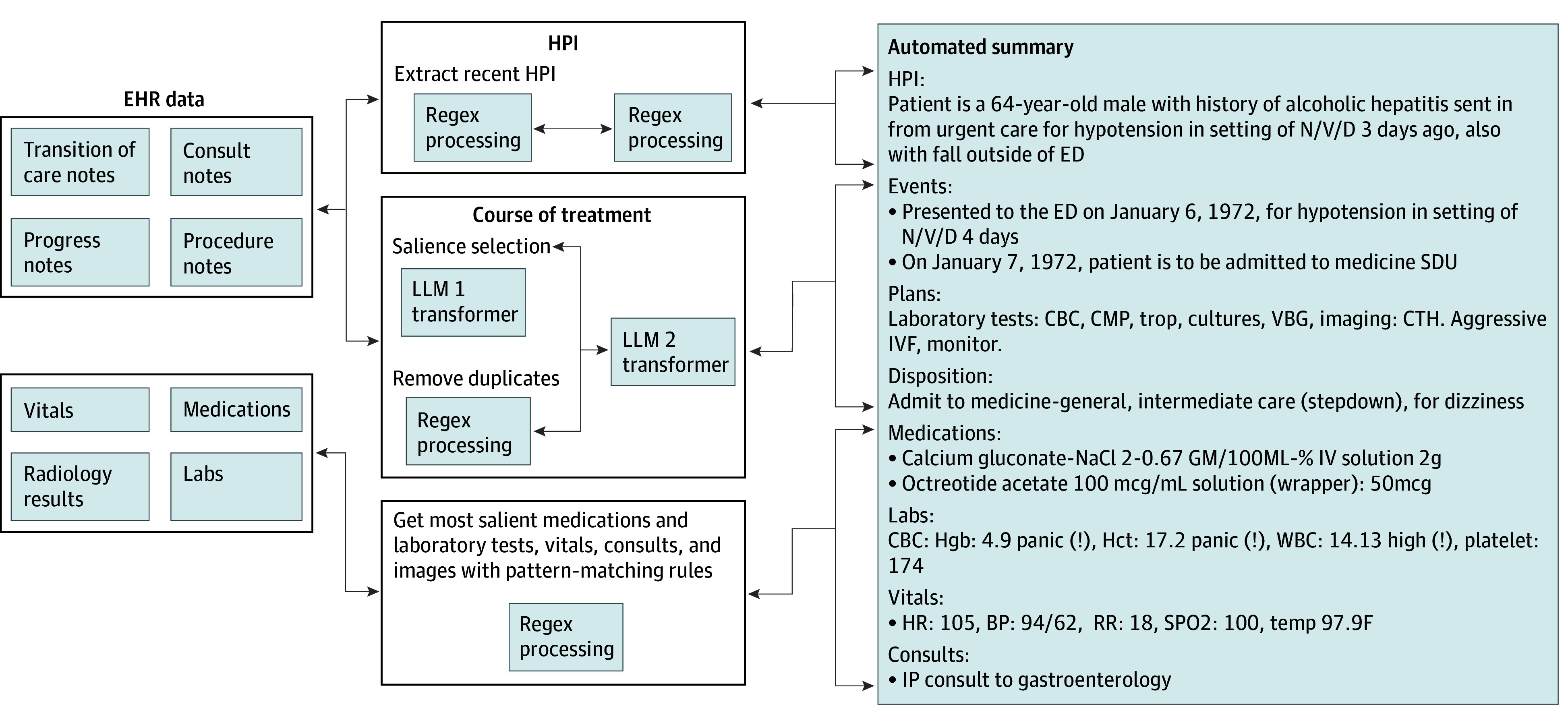
Data Flow of Generating Emergency Department (ED) Handoff Summary CBC indicates complete blood count; CMP, comprehensive metabolic panel; CTH, computed tomography of the head; EHR, electronic health record; Hct, hematocrit; Hgb, hemoglobin; HPI, history of present illness; HR, heart rate; IP, inpatient; IVF, intravenous fluid; N/V/D, nausea, vomiting, and diarrhea; RR, respiratory rate; SDU, step down unit; SPO2, peripheral capillary oxygen saturation; WBC, white blood cell; WBG, whole blood glucose.

### Evaluation

It is critical to ensure that AI systems are safe, ethical, and without bias in the clinical domain. For the proposed approach, we performed comprehensive automatic evaluations and a novel, rigorous, patient safety-focused clinical evaluation. The unique clinical evaluation framework was designed to (1) screen for and identify the common, specific correctness issues in LLMs observed in longform clinical summarization and (2) assess the potential patient safety implications associated with any incorrectness identified using a modified version of the World Health Organization’s International Classification for Patient Safety.^45^

### Automated Evaluations

We used the summarization evaluation metrics of recall-oriented understudy for gisting evaluation (ROUGE),^46^ bidirectional encoder representations from transformers score (BERTScore),^47^ and source chunking approach for large-scale inconsistency evaluation (SCALE).^48^ ROUGE computes the overlap of n-grams between the generated and reference summaries. For longform document summarization, the following ROUGE scores are considered to be close to the reference summaries: ROUGE-1, above 0.4; ROUGE-2, above 0.2; and ROUGE-L, above 0.3.^46^ BERTScore leverages the pretrained contextual embeddings from BERT and matches words to compute a similarity score for each token in the candidate sentence with each token in the reference sentence. We used SCALE,^48^ a natural language inference–based approach, to measure the faithfulness between the source document and the generated text. Further background is provided about SCALE in eAppendix 2 in Supplement 1.

### Statistical Analysis

Based on prior work, 3 board certified EM physician leaders (M.M., A.F., and P.S.) with experience in formal quality and patient safety review processes performed retrospective reviews of ED-based EHR records of 50 individual ED patient encounters, randomly selected from the test dataset.^49^ Based on prior published clinical evaluations of LLM, as well as the study feasibility of using EM physician quality and patient safety leaders, 50 ED patient encounters were evaluated.^50^ Reviewers subsequently evaluated 2 ED-to-inpatient handoff notes for each patient: (1) the physician-written note and (2) the LLM-generated note.

On a Likert scale of 1 to 5, where 1 is unacceptable and 5 is excellent, the 3 physicians rated the completeness, curation, readability, and correctness of the summary as shown in eTable 1 in Supplement 1. Physicians rated the usefulness of the summary, defined as the capability of the summary being incorporated into a workflow where a physician would make edits before final completion, mitigating potential future self-referential learning loops and the downstream adverse consequences.^51^ Likewise, the raters assessed potential patient safety implications of unmitigated model errors using a scale from 1 to 5, where 1 denotes life-threatening risks and 5 denotes no identified patient safety risk for completeness, curation, readability, and the 4 subcategories within correctness (hallucination, faulty logic, knowledge gap, and bias), as well as the overall patient safety risk.^45^ Evaluators arrived at prestudy consensus that a usefulness Likert score of at least a 3 out of 5 indicated that the LLM-generated summary likely demonstrated baseline acceptability for such a workflow. To extrapolate a theoretical worst case scenario, the physicians rated the safety of the LLM-generated summary as defined as the capability of the summary to fully replace a physician-written note (unmitigated).

To improve consistency and agreement, the 3 reviewers met to familiarize themselves with the framework and evaluated 10 separate cases from the test dataset that were not included in the clinical evaluation results. Additionally, after independently scoring the summaries, they met to ensure consensus interpretation of the multidimensional scoring framework. Interrater reliability was calculated using intraclass correlation coefficient (ICC), using a 2-way random effects model for consistency with the Pingouin statistical package version 0.5.4 in Python (Python Software Foundation). The ICC measures the similarity of the 3 raters to confirm the consistency and validity of the evaluation protocol; the scores are from 0 to 1, where 1 indicates unanimous agreement and 0 represents no agreement.^52^ Data were analyzed from October 2023 to March 2024.

## Results

### Automated Tasks

Of 1600 patients, the mean (SD) age was 59.8 (18.9) years and 832 (52%) were female. In Table 2, ROUGE and BERTScore compare the summaries with the testing set from our annotations, and SCALE score compares the summaries with the source notes. From automatic evaluation results, we observed that LLM-generated summaries had better scores than the physician summaries, such that ROUGE-2 was 0.322 vs 0.088, BERT-precision was 0.859 vs 0.796, and SCALE was 0.691 vs 0.456, suggesting the LLM-generated summaries were more similar and more detailed than the physician summaries.

**Table 2.** Automated Evaluation Scores, Large Language Model (LLM)–Generated and Physician-Written

Summary type	R-1^a^	R-2^a^	R-L^a^	BERT-p	BERT-r	SCALE
LLM-generated	0.494	0.322	0.391	0.859	0.876	0.691
Physician-written	0.251	0.088	0.154	0.796	0.827	0.456

Abbreviations: BERT, bidirectional encoder representations from transformers; p, precision-based scores; r, recall-based scores; R, recall-oriented understudy for gisting evaluation; SCALE, source chunking approach for large-scale inconsistency evaluation.

^a^ R-1, R-2, R-L are the 3 types of recall-oriented understudy for gisting evaluation scores. Higher is better for all metrics.

### Clinical Evaluation Tasks

The clinical evaluation results for LLM-generated summaries and physician-written summaries are shown in Table 3 and Table 4. The mean clinical quality scores of the automated summaries are in a comparable range (4-5) to those of the physician summaries. However, the automated summaries were observed to be of lower quality compared with the physician-written summaries with regards to mean (SD) usefulness (4.04 [0.85] vs 4.36 [0.71]), completeness (4.00 [0.88] vs 4.16 [0.84]), curation (4.24 [0.58] vs 4.76 [0.48]), readability (4.00 [0.64] vs 4.64 [0.49]), correctness (4.52 [0.64] vs 4.90 [0.39]), and patient safety (4.06 [0.86] vs 4.50 [0.56]).

**Table 3.** Mean Clinical Quality Evaluation, Large Language Model (LLM)–Generated and Physician-Written

Criteria	LLM-generated						Physician-written					
	Mean score (SD)	Likert rating 1-5, No. (%)^a^					Mean score (SD)	Likert rating 1-5, No. (%)^a^				
		1	2	3	4	5		1	2	3	4	5
Completeness	4.00 (0.88)	0	12 (8)	31 (20.7)	69 (46)	38 (25.3)	4.16 (0.84)	0	3 (2)	31 (20.7)	48 (32)	68 (45.3)
Curation	4.24 (0.58)	0	1 (0.7)	13 (8.7)	85 (56.7)	51 (34)	4.76 (0.48)	0	0	6 (4)	39 (26)	105 (70)
Readability	4.00 (0.64)	0	8 (5.3)	17 (11.3)	87 (58)	38 (25.3)	4.64 (0.49)	0	0	5 (3.3)	38 (25.3)	107 (71.3)
Correctness	4.52 (0.64)	0	0	13 (8.7)	39 (26)	98 (65.3)	4.90 (0.39)	0	0	2 (1.3)	12 (8)	136 (90.7)
Usefulness	4.04 (0.86)	0	12 (8)	30 (20)	59 (39.3)	49 (32.7)	4.36 (0.71)	0	5 (3.3)	13 (8.7)	53 (35.3)	79 (52.7)

^a^ Likert scores and score distributions over 50 notes for 3 annotators. There are no 1 ratings for either physician or LLM summaries in the 150 evaluation results.

**Table 4.** Mean Clinical Safety Evaluation, Large Language Model (LLM)–Generated and Physician-Written

Criteria	LLM-generated						Physician-written					
	Mean (SD)	Likert score 1-5, No. (%)^a^					Mean (SD)	Likert score 1-5, No. (%)^a^				
		1	2	3	4	5		1	2	3	4	5
Completeness	4.20 (0.93)	0	13 (8.7)	19 (12.7)	58 (38.7)	60 (40)	4.50 (0.65)	0	0	17 (11.3)	43 (28.7)	90 (60)
Curation	4.82 (0.32)	0	1 (0.7)	3 (2)	21 (14)	125 (83.3)	4.90 (0.31)	0	0	3 (2)	8 (5.3)	139 (92.7)
Readability	4.74 (0.37)	0	1 (0.7)	6 (4)	23 (15.3)	120 (80)	4.94 (0.14)	0	0	0	10 (6.7)	140 (93.3)
Correctness: hallucination	4.96 (0.14)	0	0	0	5 (3.3)	145 (96.7)	5.00	0	0	0	0	150 (100)
Correctness: knowledge gap	4.88 (0.48)	0	3 (2)	2 (1.3)	6 (4)	139 (92.7)	4.90 (0.42)	0	1 (0.7)	5 (3.3)	3 (2)	141 (94)
Correctness: faulty logic	4.60 (0.75)	0	11 (7.3)	12 (8)	13 (8.7)	114 (76)	4.94 (0.24)	0	0	2 (1.3)	2 (1.3)	146 (97.3)
Correctness: bias	5.00	0	0	0	0	150 (100)	5.00	0	0	0	0	150 (100)
Overall safety risk	4.06 (0.86)	0	11 (7.3)	27 (18)	60 (40)	52 (34.7)	4.50 (0.56)	0	1 (0.7)	16 (10.7)	41 (27.3)	92 (61.3)

^a^ Likert scores and score distributions over 50 notes for 3 annotators. There are no 1 ratings for either physician or AI summaries in the 150 evaluation results.

In extrapolating the estimated worst-case scenario impact of these performance gaps on patient safety, the 3 expert clinicians determined none of the identified model performance issues were anticipated to create a level 1 (life-threatening) safety event (see examples of worst case scenarios in eTable 2 in Supplement 1). While the incompleteness and faulty logic identified in the automated summaries received mean (SD) safety scores of 4.20 (0.93) and 4.60 (0.75), respectively; 13 (8.7%) and 11 (7.3%) of these events, respectively, were determined to have the potential to create a level 2 patient safety event following EM-to-IP handoff, substantially higher compared with the physician-written summaries (0%). All of the 5 hallucinations had patient safety scores between 4 and 5 and a mean (SD) score of 4.96 (0.14), which is defined as the hallucinations posing mild to no patient safety risk. LLM-generated notes demonstrated a higher rate of incorrectness (9.6%) compared with the physician-written notes (2.0%), although very few hallucinations.

ICC were 0.79 for completeness, 0.70 for curation, 0.59 for readability, 0.76 for correctness, and 0.74 for usefulness. These numbers suggest good reliability of agreement for completeness, curation, correctness, and usefulness and suggest fair reliability for readability among the 3 raters.

## Discussion

The study demonstrated success in generating EM-to-IP handoff notes using both a fine tuned, pretrained LLM and rule-based approaches within an end user–developed note template. It is important to note that (largely due to time constraints within the EM care delivery model) the performance of EM-to-IP handoff notes was not the current standard of care in EM. The study site’s unique electronic handoff process enabled a comparison between physician-written and LLM-generated handoff notes. Traditional automated evaluations of the model output suggested superior performance. However, while the manual clinical evaluation demonstrated the majority of the LLM-generated notes were of promising comparative quality (scores of 4-5), they were, on average, inferior to the clinician-written notes.

Our novel clinical evaluation’s findings suggest the majority of identified quality limitations and incorrectness would have minimal impact on patient safety, even when extrapolated to the worst-case scenario of the LLM-generated summary content not being reviewed and edited by a clinician before completion. This was designed to address contemporary LLM concerns of user trust, reliance and expertise.^49^ As such, none of the incorrect output text elements reached life-threatening risk. However, incompleteness and faulty logic identified in the automated summaries were not always negligible, with just under 1 in 10 of these performance gaps determined to have the potential to create significant patient safety risk compared with the physician-written summaries. These critical implementation safety findings will inform (1) directionality of further model refinement; (2) further clinical evaluation of postrefinement model output; and (3) irrespective of downstream model performance, an EHR-implementation plan constrained to a user-interface design that will allow EM clinicians to review and edit the LLM-generated handoff note as a draft before finalizing (see eAppendix 1 in Supplement 1). This physician-in-the-loop process has also been identified as critical in other recent work implementing LLMs into clinical workflows.^29,53^

While the automated methods of SCALE and MPNet-based sentence transformers demonstrated a cursory view of the faithfulness performance of the models, the clinical evaluation provided the nuanced context of the true factuality of our system on a word by word level. When comparing with the source notes, the automatic evaluations rewarded the summaries with more details, more semantic similarities, and more entailment logics, while physician-written notes tended to be more concise with more shortcuts and clinical jargon, which are penalized by automatic evaluation metrics. In addition, LLM-generated summaries are completely based on the source notes, while physician-written summaries are often composed with additional knowledge that cannot be found from the source notes.

The divergence of the automated and clinical evaluation results of an LLM intended for integration into a critical clinical workflow is an important finding. First, this observed finding validates the importance of clinical evaluations in addition to conventional automated evaluations to determine accuracy.^54^ While other LLM clinical evaluation frameworks have been described to measure conventional model output quality categories (such as incorrectness domains and other performance gaps),^30,35^ to our knowledge, our novel framework is the first to incorporate anticipated patient safety implications for each individual category deficiency.

### Limitations

There were several limitations to the study that were primarily driven from constraints of infrastructure, as well as regulations, legal governance, and labor requirements. At the study location, the data were required to remain on premise at all times and the infrastructure that was provided had a GPU limitation of 24 GB. Given these infrastructure restrictions, the best open-source model available during the study was LLM 2. Furthermore, we were not able to demonstrate the comparable difference between our fine-tuned LLM 2 model and third party LLMs^32,55^ because of the study location’s restrictions and concerns with the data retention policies. Nevertheless, our study demonstrates the potential capability of integrating state-of-the-art open source LLMs at organizations that are less open to integrating third-party LLMs.

While the dataset was smaller, we made significant efforts to reduce model variance and prevent overfitting by allocating more data to the training cohort and using k-fold cross validation. And while our ratio split choice implies the testing results will have slightly greater variance than expected, this is mitigated through the extensive manual clinical assessment that was performed. The study’s multidimensional clinical evaluation was labor intensive, requiring more than 200 hours from expert informaticists and quality trained clinician experts to both curate the dataset of 1600 records and perform manual comparative evaluations of 50 LLM-generated and 50 clinician-generated summary notes within the context of complex ED encounters. This approach is unlikely scalable, invoking complex postimplementation governance questions that remain unanswered in the medical literature and invoke the need for future research focused on the possibility of AI performing the clinical evaluations.^56^ Lastly, the relatively infrequent but potentially significant patient safety implications of model output incorrectness and incompleteness warrants further model refinement and repeat clinical evaluation, as described in the eAppendix 1 in Supplement 1 overview of our rigorous preimplementation model development and testing framework.

## Conclusions

This study’s results suggest promise for future thoughtful integration of LLM-generated EM-to-IP handoff notes into clinical admission workflows, as well as the associated potential downstream quality and efficiency gains. Our novel clinical evaluation framework demonstrates an effective preimplementation strategy to measure potential patient safety implications of incorrectness identified in LLM-generated clinical care summaries, which will guide future model refinement and implementation strategies. In the absence of a current written standard of care in EM, this innovation could represent a transformative advancement in the quality of EM-to-IP transitions of care.

## Notes

Supplement 1. **eAppendix 1.** Technology Specifications, Model Training, and Inference **eAppendix 2. **Automated Method of SCALE **eTable 1.** Definitions From the Clinical Assessment **eTable 2. **Worst Case Scenario of Incorrectness Examples From LLM-Generated Notes

Supplement 2.
Data Sharing Statement
