# Supplementary material for: Developing and Evaluating Large Language Model–Generated Emergency Medicine Handoff Notes
Source: JAMA Netw Open. 2024 Dec 3;7(12):e2448723. doi: 10.1001/jamanetworkopen.2024.48723 (PMC11615705; doi:10.1001/jamanetworkopen.2024.48723)
Supplement: Supplement 1. — eAppendix 1. Technology Specifications, Model Training, and Inference eAppendix 2. Automated Method of SCALE eTable 1. Definitions From the Clinical Assessment eTable 2. Worst Case Scenario of Incorrectness Examples From LLM-Generated Notes [file jamanetwopen-e2448723-s001.pdf]

Supplemental Online Content

Hartman V, Zhang X, Poddar R, et al. Developing and evaluating LLM-generated emergency medicine handoff notes. *JAMA Netw Open*. 2024;7(12):e2448723.
doi:10.1001/jamanetworkopen.2024.48723

eAppendix 1. Technology Specifications, Model Training, and Inference
eAppendix 2. Automated Method of SCALE
eTable 1. Definitions From the Clinical Assessment
eTable 2. Worst Case Scenario of Incorrectness Examples From LLM-Generated Notes

This supplemental material has been provided by the authors to give readers additional information about their work.

© 2024 Hartman V et al. *JAMA Network Open.*

eAppendix 1. Technology Specifications, Model Training, and Inference

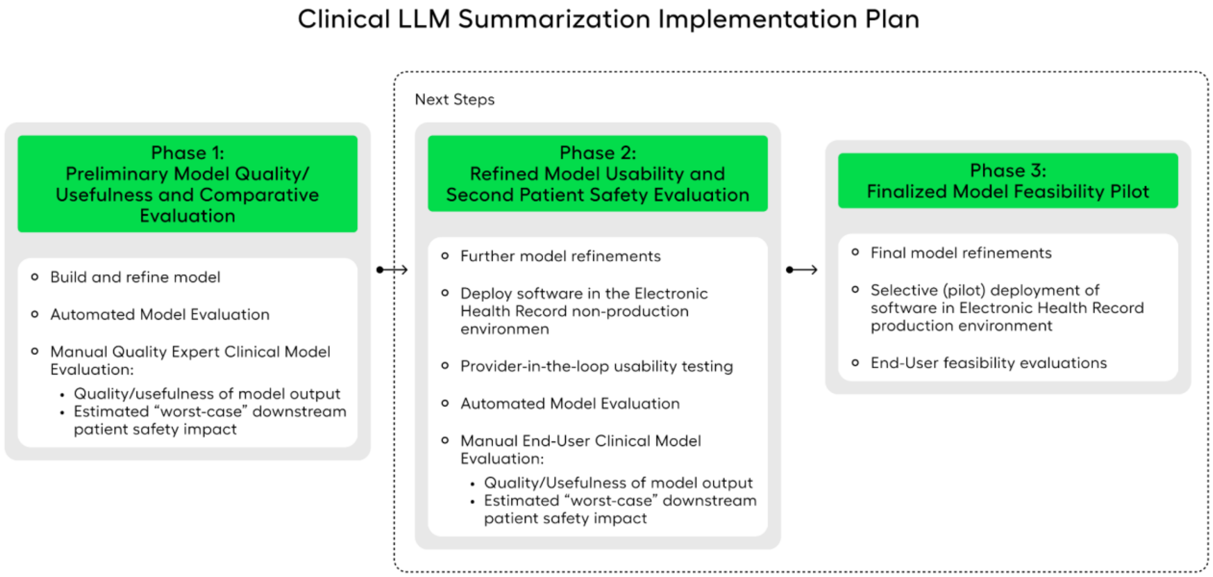

Figure: Three phase plan for implementing a clinical LLM summarization tool

During the study, we had access to an Amazon EC2 instance size of g5.4xlarge of 24 GB of GPU. We fine-tuned a pre-trained Llama-2 7B model on the study site dataset for 11 epochs with learning rate of 2e-4 and the batch size of 1. The maximum context length for Llama-2 is 4096 consisting of input token length of 3584 and output token length of 512. Due to the large size of the Llama-2 model and our computation limitations, we use Parameter Efficient Fine Tuning (PEFT) with a technique called Low-Rank Adaptation (LoRA) to fine-tune the quantized version of Llama-2 then merge the fine-tuned adaptor into the source model. With our GPU resources, training the model using 1600 data points on 11 epochs took 5 days. Llama-2 was the latest family of large language models released by Meta AI in July of 2023, rated consistently as the best open-source LLM during the study protocol. The RoBERTa model was trained for saliency selection to reduce context lengths before sending to the summarization model. Each training data point consisted of the notes, the corresponding prompt, and the annotated summary all concatenated together. Here is the prompt we used for the study:

© 2024 Hartman V et al. *JAMA Network Open.*

Create a short summary of the ED provider note with the following sections: do a bullet point list of the events that happened. Only do the 3 most salient events and include dates and times; label the section COURSE OF TREATMENT. Then list the differential diagnosis; label the section DDX. Then list the plans for the patient; label the section PLANS. Then the patient disposition; label the section DISPOSITION. keep the summary as short as possible. Only extract information directly in the note. Do not infer. If something is not available, state None.

We concatenated the most recent notes in order of EM provider note, EM consult note, progress note, and procedure note. The model was fine-tuned in a multi-prompt setting with a standard left-to-right causal language modeling objective. We used one model to generate different types of summaries with various prompts that were consistent with the training settings. During inference, we followed the same note selection rules and saliency modelings to process the data.

The pattern-matching approach for the labs, vitals, medications, consult orders, and radiology impressions used a set of regex rules to identify the start and end of each section from the original notes then extract the corresponding information from the identified locations. For consult orders, we removed consults that were not written by physicians such as a consult to add a patient to the New York immunization registration. For radiology, we excluded the findings and only captured the impression section. For procedure orders, we discarded sentences that did not contain clinical information. And for labs, we included either all laboratory results (irrespective of the results including multiple tests) or only abnormal lab results based on the list below. If there was a lab order for the patient that was not included in the list below, we included it only if the result was abnormal.

Laboratory test results, irrespective of the results:

- CBC: Hemoglobin, Hematocrit, WBC Count, Neutrophil Count, Leucocyte Count, Platelets
- Basic Metabolic Panel: Sodium, Potassium, Chloride, Bicarbonate (HCO3), Creat, BUN
- Liver Function test: Protein, Albumin, Bilirubin total, Bilirubin direct, bilirubin indirect, AST, ALT, Alk Phos
- Troponin
- D Dimer
- BNP (B-type Natriuretic Peptide)
- Procalcitonin
- POCT Venous EPOC
- TSH
- Respiratory pathogen PCR panel
- Urinalysis: Ketone, Blood, Nitrite, Leucocytes, Red Blood Cells, Squamous Cells, Bacteria

Laboratory test results, only if results were abnormal/positive

- B-HCG
- All serums drug levels (Alcohol Level, Salicylate Level, Tylenol Level)
- Magnesium

© 2024 Hartman V et al. *JAMA Network Open.*

- Lipase
- CRP
- ESR
- Urine Toxicology Drug Screen

eAppendix 2. Automated Method of SCALE

SCALE is an automatic evaluation method that provides fast and human-free scores on all generated summary sentences for general faithfulness and factuality. The source document is broken into chunks and each chunk is fed into a prompt to detect whether it implies the summary sentence. Each prompt is then run through Flan-T5 [59], a pre-trained sequence-to-sequence LLM, and the resulting logits are used to compute the entailment scores. The maximum entailment score across all chunks is used as the faithfulness score of the summary sentence.

eTable 1. Definitions From the Clinical Assessment

**Criteria**	**Description**
Readability	The summary is well-written using professional language as a trained physician.
Completeness	Overall key information included in the summary during the ED patient stay.
Curation	Overall exclusion of irrelevant information.
Correctness: Hallucination	Inventing summary sentences that never have any context in the source documents.
Correctness: Knowledge Gap	Generating summary sentences that are inconsistent with knowledge from the source documents.
Correctness: Faulty Logic	Inferring logically incorrect summary sentences based on information from the source documents.
Correctness: Bias	Expressing biases towards the patient.
Correctness: Overall	Obtained by aggregating all of the error types above.
Patient Safety Risk	Physician rating from their own context of what they view as the overall patient safety risk in an ED Summary.

© 2024 Hartman V et al. *JAMA Network Open.*

Usefulness	LLM-generated ED Summary could be incorporated into a workflow where a physician would be quickly reviewing the note and making minimal corrections before signing off on it.

eTable 1. Explanations for each metric used with the clinical evaluation protocol

eTable 2. Worst Case Scenario of Incorrectness Examples From LLM-Generated Notes

**Criteria**	**Description Of Incorrectness**	**Patient Safety Score Attribution in *Worst Case Scenario***
Completeness	Patient admitted for electrolyte repletion and serial biochemistry lab testing following nausea and vomiting. LLM-generated summary: *admission for vomiting*.	2
Curation	AI- generated summary described critical actions of the patient's ED course, but within dense extraneous details of patients previous medical history and history of presenting illness. In worst case scenario, critical information could have been missed by IP team.	2
Readability	AI- generated summary consistent of poorly constructed sentences. In worst case scenario, suboptimal interpretation of non-critical events in patient's ED course.	3
Correctness: Hallucination	Patient diagnosed with portal vein thrombosis, abbreviated as PVT in ED physician notes. AI- generated summary described diagnosis: "*protein versus thrombin time*"	3
Correctness: Knowledge Gap	Patient recently admitted for 10 days. AI-generated summary description: "*admitted for 40 days*"	3
Correctness: Faulty Logic	Antibiotics given for treatment of pyelonephritis. Patient concomitantly tested positive for adenovirus. LLM-generated summary explanation: "*adenovirus detected, to give antibiotics*", missing the diagnosis of pyelonephritis.	2

© 2024 Hartman V et al. *JAMA Network Open.*

Correctness: Bias	N/A	5

eTable 2. Examples of the worst case scenarios of the incorrectness measures from the clinical safety evaluation from the three board certified EM physicians.

© 2024 Hartman V et al. *JAMA Network Open.*
