# Supplementary material for: Developing and Evaluating Large Language Model–Generated Emergency Medicine Handoff Notes
Source: JAMA Netw Open. 2024 Dec 3;7(12):e2448723. doi: 10.1001/jamanetworkopen.2024.48723 (PMC11615705; doi:10.1001/jamanetworkopen.2024.48723)
Supplement: Supplement 2. — Data Sharing Statement [file jamanetwopen-e2448723-s002.pdf]

Data Sharing Statement

Hartman. Developing and Evaluating Large Language Model–Generated Emergency Medicine Handoff Notes. *JAMA Netw Open*. Published December 03, 2024. doi:10.1001/jamanetworkopen.2024.48723

Data
Data available: Yes
Data types: Participant data with identifiers
How to access data: Request for data will be made available upon request to Vince Hartman by email to vince@abstractivehealth.com.
When available: With publication

Supporting Documents
Document types: Statistical/analytic code
How to access documents: Request for the statistical code will be made available upon request to Vince Hartman by email to vince@abstractivehealth.com.
When available: With publication

Additional Information
Who can access the data: Researchers whose proposed use of the data has been approved by Weill Cornell Medicine/NewYork Presbyterian
Types of analyses: Researchers whose proposed use of data is for the advancement of LLM clinical summarization.
Mechanisms of data availability: Data will be made available after a signed data access agreement.
